# Case report: Primary CDK4/6 inhibitor and endocrine therapy in locally advanced breast cancer and its effect on gut and intratumoral microbiota

**DOI:** 10.3389/fonc.2024.1360737

**Published:** 2024-03-27

**Authors:** Guilherme Vilhais, Diogo Alpuim Costa, Mário Fontes-Sousa, Pedro Casal Ribeiro, Filipa Martinho, Carolina Botelho de Sousa, Catarina Rodrigues Santos, Ida Negreiros, Ana Canastra, Paula Borralho, Ana Guia Pereira, Cristina Marçal, José Germano Sousa, Renata Chaleira, Júlio César Rocha, Conceição Calhau, Ana Faria

**Affiliations:** ^1^ Haematology and Oncology Department, CUF Oncologia, Lisbon, Portugal; ^2^ NOVA Medical School (NMS), Faculdade de Ciências Médicas (FCM), Universidade NOVA de Lisboa (UNL), Lisbon, Portugal; ^3^ Medical Oncology Department, Hospital de Cascais, Cascais, Portugal; ^4^ Breast Unit, CUF Oncologia, Lisbon, Portugal; ^5^ Bioinformatics Department, Centro de Medicina Laboratorial Germano de Sousa, Lisbon, Portugal; ^6^ Surgery Department, Instituto Português de Oncologia de Lisboa Francisco Gentil (IPOL-FG), Lisbon, Portugal; ^7^ Anatomic Pathology Department, CUF Oncologia, Lisbon, Portugal; ^8^ Institute of Anatomic Pathology, Faculdade de Medicina da Universidade de Lisboa (FMUL), Lisbon, Portugal; ^9^ Genetics Laboratory, Centro de Medicina Laboratorial Germano de Sousa, Lisbon, Portugal; ^10^ Clinical Pathology Department, Centro de Medicina Laboratorial Germano de Sousa, Lisbon, Portugal; ^11^ Psychology Department, CUF Descobertas Hospital, Lisbon, Portugal; ^12^ Centro de Investigação em Tecnologias e Serviços de Saúde (CINTESIS), Rede de Investigação em Saúde (RISE), NOVA Medical School (NMS), Faculdade de Ciências Médicas (FCM), Universidade NOVA de Lisboa (UNL), Lisbon, Portugal; ^13^ Unidade Universitária Lifestyle Medicine José de Mello Saúde, NOVA Medical School (NMS), Lisbon, Portugal; ^14^ Comprehensive Health Research Centre (CHRC), NOVA Medical School, Faculdade de Ciências Médicas, Lisbon, Portugal

**Keywords:** breast cancer, CDK 4/6 inhibitors, gut microbiota, gut microbiome, microbiota, microbiome, intratumoral microbiota

## Abstract

Locally advanced breast cancer poses significant challenges to the multidisciplinary team, in particular with hormone receptor (HR) positive, HER2-negative tumors that classically yield lower pathological complete responses with chemotherapy. The increasingly significant use of CDK 4/6 inhibitors (CDK4/6i) plus endocrine therapy (ET) in different breast cancer settings has led to clinical trials focusing on this strategy as a primary treatment, with promising results. The impact of the microbiota on cancer, and vice-versa, is an emerging topic in oncology. The authors report a clinical case of a postmenopausal female patient with an invasive breast carcinoma of the right breast, Luminal B-like, staged as cT4cN3M0 (IIIB). Since the lesion was considered primarily inoperable, the patient started letrozole and ribociclib. Following 6 months of systemic therapy, the clinical response was significant, and surgery with curative intent was performed. The final staging was ypT3ypN2aM0, R1, and the patient started adjuvant letrozole and radiotherapy. This case provides important insights on primary CDK4/6i plus ET in locally advanced unresectable HR+/HER2- breast cancer and its potential implications in disease management further ahead. The patient’s gut microbiota was analyzed throughout the disease course and therapeutic approach, evidencing a shift in gut microbial dominance from Firmicutes to Bacteroidetes and a loss of microbial diversity following 6 months of systemic therapy. The analysis of the intratumoral microbiota from the surgical specimen revealed high microbial dissimilarity between the residual tumor and respective margins.

## Introduction

Breast cancer is the second most common neoplasm worldwide and represents the leading cause of cancer-related death among women in over 100 countries (1). It is a heterogeneous disease that can be further classified into different molecular subtypes with specific prognostic and therapeutic implications. Hormone receptor-positive (HR+) and HER2-negative (HER2-) breast cancer is the most common subtype, accounting for more than 65% of all breast cancers (2).

Endocrine therapy (ET) is the mainstay of HR+/HER2- breast cancer’s systemic therapy, being recommended as adjuvant treatment in early disease and as the preferred option in the metastatic setting in the absence of visceral crisis (3–5). However, in recent years, this disease’s systemic approach has changed considerably with the discovery and establishment of CDK 4/6 inhibitors (CDK4/6i), initially in the metastatic setting and, more recently, in adjuvancy (6–8). The cyclin-dependent kinases (CDKs) are a large family of serine-threonine kinases that have important roles in cell cycle regulation (9). The dysregulation of mechanisms that govern the cell cycle, such as the complex interplay between cyclins and their associated CDKs, results in uncontrolled cellular proliferation and constitutes one of the hallmarks of cancer (10). Cyclin D binds CDK 4/6 and then hyperphosphorylates retinoblastoma protein (pRb), which results in cancer cell cycle progression. CDK4/6i block the hyperphosphorylation of pRb, causing G1 arrest and thereby hindering proliferation (11).

The increasing evidence that complex microbial ecosystems play a substantial role in tumorigenesis, cancer differentiation, and malignant progression has recently led to the inclusion of polymorphic microbes as an emerging hallmark of cancer (12). The most significant evidence for this integrated role comes from studying microbes within the gastrointestinal tract, also known as gut microbiota. However, there has been a growing appreciation of the role of these polymorphic microbes in other tissues and organs, including those living within tumors (intratumoral microbiota).

Gut microbiota is unique in each individual and is determined by lifestyle and genetic factors, posing a challenge in distinguishing healthy from abnormal gut microbiota. Microbial dysbiosis refers to a maladaptation or abnormal composition of the microbial community of a given organ or tissue, and increasing evidence suggests it may influence tumor biology, drug metabolism, and immune system regulation (13). To understand the differences between homeostatic and dysbiotic microbiota, it is essential to comprehend the concepts of α and β-diversity (14). α-diversity measures the diversity of microbial species within a sample and can be calculated by Operational Taxonomic Units (OTUs) count (which refers to the number of different species in the sample) or by Simpson’s and Shannon’s diversity indices (which measure how evenly the microbes are distributed). β-diversity is used to compare different samples, accessing the differences in microbial composition between them.

It is believed that both gut and breast microbiota may play a role in breast carcinogenesis, namely through the secretion and metabolism of hormone-like bioactive compounds (14, 15). Intratumoral microbiota is thought to contribute to cancer initiation and progression through DNA mutations, activation of carcinogenic pathways, promotion of chronic inflammation, the complement system, initiation of the metastatic process, and modulation of antitumor immunity (16). Different tumor types have distinct intratumoral microbial compositions, with breast cancer standing out for a particularly rich and diverse microbiota (17).

The prognostic value of gut and intratumoral microbiota in breast cancer is an active research area. Several studies have found correlations between specific microbiota compositions and outcomes such as tumor progression, metastases, response to therapy, and toxicities (14, 18–20). However, the clinical significance of these findings remains mainly unclear, and microbiota analysis and modulation strategies are not current practice in breast cancer management.

This paper depicts the clinical case of a postmenopausal female patient diagnosed with a locally advanced HR+/HER2- breast carcinoma that was considered primarily unresectable and was therefore proposed for systemic therapy with an aromatase inhibitor (AI) and a CDK4/6i, achieving a good clinical response. The case demonstrates the potential of this therapeutic approach in a setting in which high-level evidence is still lacking. Moreover, the patient was included in the BioBreast study, a study that aims to understand the interplay between microbiota and systemic therapy in breast cancer patients, allowing a unique analysis of the patient’s gut microbiota throughout the disease course and therapeutic approach, as well as a comprehensive characterization of intratumoral microbiota.

## Case description

### Patient information

A female patient in her late 60s consulted a general surgeon because of a painful mass in her intermammary cleft that lasted for approximately four months. The patient’s medical history revealed essential hypertension and dyslipidemia, and she was medicated accordingly with olmesartan and simvastatin. Her surgical history revealed a previous ovarian cystectomy. Her menarche was at 17 years old, menopause at 50, and she had two pregnancies, two deliveries (G2P2A0) and breastfed. The patient did not report any family history of breast, uterine, or ovarian cancer.

### Clinical findings

**Figure 1:**
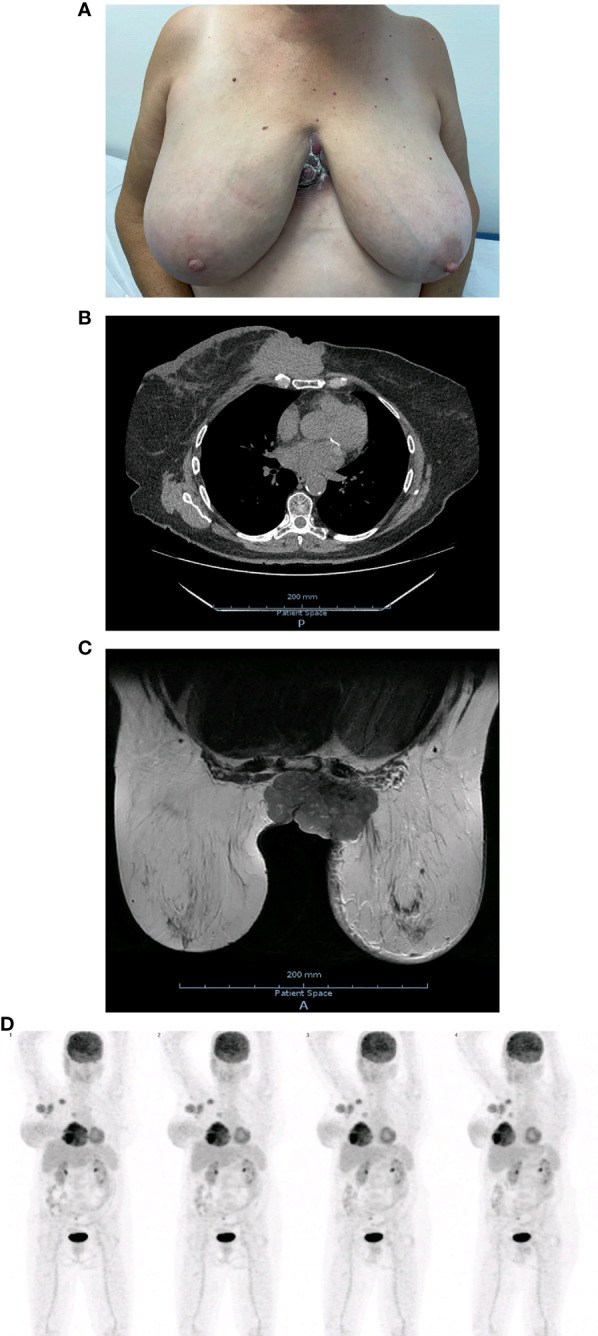
**(A)** Initial clinical presentation. **(B)** Initial chest CT. **(C)** Initial breast MRI. **(D)** Whole body on PET/CT with 18F-FDG at initial staging.

At physical examination, she presented an enlarged right breast with lower-quadrant edema and an infiltrative ulcerated mass with a multinodular aspect in the intermammary cleft (**Figure 1A**). At palpation, it was possible to identify multiple right axillary adenopathies.

### Timeline

**Figure:**
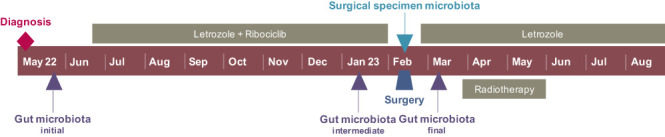


### Diagnostic assessment

Following this presentation, she underwent an ultrasound-guided core biopsy of the mass and a chest computerized tomography (CT). The chest CT revealed a solid mass with irregular borders in the medial area of the right breast measuring 53 x 82 x 86 mm with posterior involvement of the sternum and chondrosternal joints (**Figure 1B**) and multiple enlarged nodes in the right axillary region. The core biopsy confirmed an invasive breast carcinoma of no special type (NST), moderately differentiated (Grade 2), GATA3+, CK7+, with an expression of estrogen and progesterone receptors in 95% and 75% of tumor nuclei, respectively. The C-ERB-B2 score was 1+, and the tumor’s proliferative index, accessed by Ki67 expression, was 30%.

In order to complete a mammary study, a breast magnetic resonance imaging (MRI), ultrasound, mammogram, and an ultrasound-guided microbiopsy of the axilla were requested. The breast MRI revealed a lesion of 90 x 55 x 76 mm occupying the totality of the lower-inner quadrant of the right breast, with a necrotic component that invaded and ulcerated the overlying skin, upper anterior abdominal wall, chest wall, and the lower-inner quadrant of the left breast (**Figure 1C**). The MRI and ultrasound further revealed a suspicious lymph node in the right internal mammary chain, as well as multiple enlarged lymph nodes on levels I, II and III of the right axilla. The axillary histology revealed fibroadipose tissue infiltrated with breast carcinoma of NST without any identifiable lymph node tissue.

Positron emission tomography (PET)/CT with 18F-fluorodeoxyglucose (18F-FDG) was performed to complete staging and evaluate for possible signs of distant metastases. The exam was positive for right axillary, left parasternal, and right supraclavicular lymph nodes, without evident bone involvement or other images suggesting distant metastases (**Figure 1D**).

Considering these studies, the patient was diagnosed with invasive breast carcinoma NST of the right breast, Luminal B-like, and was staged as cT4cN3M0, corresponding to stage IIIB according to AJCC’s TNM 8^th^ edition. The patient’s case was discussed in the breast multidisciplinary meeting, and the tumor was considered locally advanced and primarily unresectable. Therefore, it was proposed to start systemic therapy with an AI with a CDK4/6i and to reassess for resectability further ahead. The patient was included in the BioBreast study, and the bacterial composition of her gut microbiota was studied by next-generation sequencing (NGS) prior to therapy initiation. Detailed information on fecal harvest and sample management is available as **Supplementary Material**.

### Therapeutic intervention

The patient started letrozole 2.5 mg once a day and ribociclib 600 mg once daily for 21 days, followed by a 7-day break to complete a 28-day treatment cycle. She underwent 6 complete cycles of ribociclib and the first two weeks of the seventh cycle. Systemic therapy was well-tolerated, with only nausea grade 1 according to Common Terminology Criteria for Adverse Events (CTCAE version 5.0) to report.

### Follow-up and outcome

**Figure 2:**
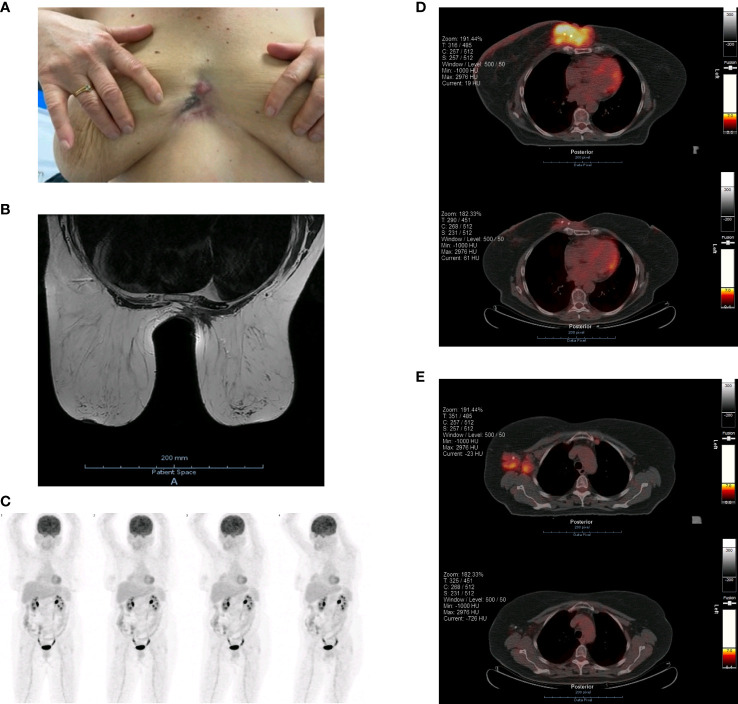
**(A)** Clinical response following 6 months of systemic therapy. **(B)** Breast MRI following 6 months of systemic therapy. **(C)** Whole body on PET/CT with 18F-FDG following 6 months of systemic therapy. **(D)** Right breast lesion on PET/CT with 18F-FDG prior to (upper image) and following 6 months of systemic therapy (lower image). **(E)** Lymph node involvement on PET/CT with 18F-FDG prior to (upper image) and following 6 months of systemic therapy (lower image).

The patient had a good local response from as early as the first cycle of ribociclib, and the lesion’s regression was very evident following six months of systemic therapy (**Figure 2A**). As part of the BioBreast study, a new sample of gut microbiota was studied following 6 months of systemic treatment. At this time, she also repeated the breast MRI and PET/CT with 18F-FDG.

The breast MRI confirmed a favorable response, with a tumor size reduction from 90 x 55 x 76 mm to 72 x 39 x 73 mm, the disappearance of the vegetation in the intermammary cleft, and suggested tumor necrosis (**Figure 2B**). The internal mammary suspicious lymph node was no longer present, and there was a significant decrease in the number and volume of the right axillary adenopathies.

The PET/CT with 18F-FDG also evidenced a favorable response compared to the previous study. There was a marked decrease in both metabolic expression (SUV_max_ 10.95 to 2.30) and tumor’s dimensions of the right breast neoplasm, as well as a complete extinction of the lymph node hypermetabolism previously documented (**Figures 2C–E**).

**Figure 3:**
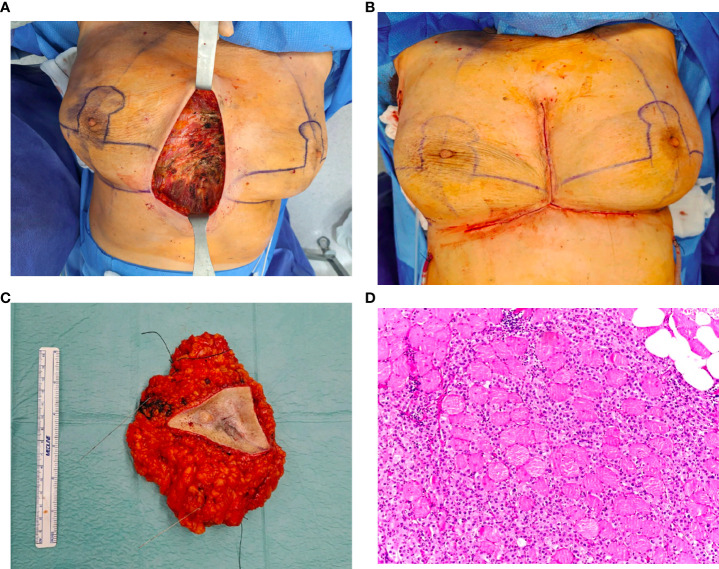
**(A)** Surgical resection of the tumor, pre- and latersternal skin, medial portions of both pectoralis muscles and part of the inner quadrants of both breasts. **(B)** Surgical reconstruction using internal mammary flaps. **(C)** Surgical specimen. **(D)** Pathological results of the surgical specimen showing tumoral infiltration of the muscular tissue.

Following this significant local response, the patient was proposed for surgery with curative intent. She underwent a resection of the previously clip-marked tumor, including pre- and latersternal skin, medial portions of both pectoralis muscles, as well as part of the inner quadrants of both breasts (**Figure 3A**). Additionally, a right axillary lymphadenectomy was performed. The posterior margin was in contact with the sternal periosteum, precluding further margin extension. The resection was followed by an immediate reconstruction using internal mammary flaps (**Figure 3B**).

The pathological results evidenced a tumor bed of 77 x 55 x 26 mm of invasive breast carcinoma NST, moderately differentiated, with infiltration of surrounding muscular tissue, adipose tissue, and superficial and deep dermis (**Figures 3C, D**). There was a 30% size reduction compared to the tumor’s original dimension. A positive posterior margin was confirmed microscopically. Following an intraoperative margin extension, the remaining margins were > 10 mm away from the tumor bed. There were 4 out of 11 axillary lymph nodes positive for metastases, none with extracapsular extension. Therefore, the tumor was restaged as ypT3ypN2aM0, R1 (posterior margin). Compared with the initial biopsy, the pathological specimen revealed estrogen receptors positivity in 50% of tumor nuclei (previously 95%); no expression of progesterone receptors (previously 75%); C-ERB-B2 score remained 1+; and the tumor’s Ki67 also remained at 30%.

As part of the BioBreast study, microbiota samples of the surgical specimen (residual tumor and respective margins) were collected and analyzed, as described in the **Supplementary Material** section.

One week after surgery, a new sample of gut microbiota was collected and studied. The case was then rediscussed in the breast multidisciplinary meeting, and the proposed plan was adjuvant radiotherapy and maintaining systemic therapy with letrozole and ribociclib. However, the patient did not intend to maintain CDK4/6i, so she continued letrozole alone and started radiotherapy. She completed image-guided radiotherapy (IGRT) using volumetric modulated arc therapy (VMAT) with conventional fractionation (28 + 7fx, 54.4Gy@1.8Gy) to the chest wall and right axillary, supraclavicular, and internal mammary lymph nodes.

**Figure 4:**
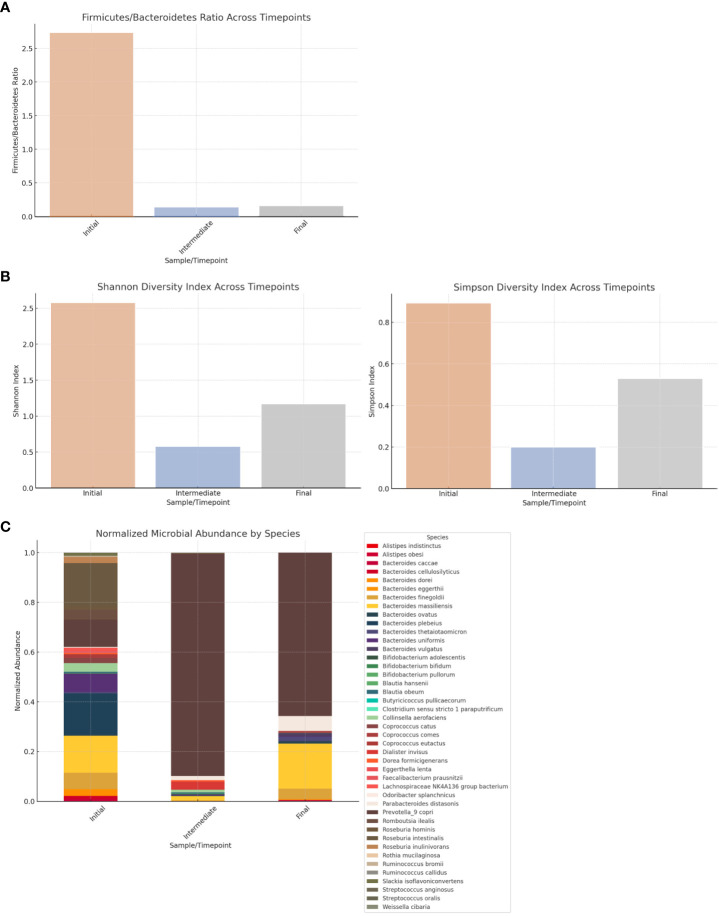
**(A)** Firmicutes/Bacteroidetes (F/B) ratio in gut microbiota across timepoints. The F/B ratio decreases from 2.73 at the initial timepoint (diagnosis) to 0.13 at the intermediate timepoint (following 6 months of systemic therapy) and 0.15 at the final timepoint (following surgery), reflecting a major shift from a Firmicutes-dominant profile at the initial timepoint to a Bacteroidetes-dominant profile in the subsequent timepoints. **(B)** α-diversity indices in gut microbiota across timepoints. At diagnosis, Shannon's and Simpson's diversity indices were 2.58 and 0.89, respectively, indicating a high diversity at this stage. There is a significant decrease in both indices at the intermediate timepoint (0.58 and 0.20), followed by a partial recovery by the final timepoint (1.17 and 0.53). **(C)** Relative abundance of microbial species in gut microbiota across timepoints.

The dynamic evolution of the gut microbiota across the three timepoints was analyzed (**Figures 4A, B**). There were statistically significant differences in microbial abundance between the three timepoints (*p*=0.015). At diagnosis, the microbial community was characterized by a notable dominance of Firmicutes phyla. Following 6 months of systemic therapy, there was a shift towards a significant prevalence of Bacteroidetes, accompanied by a marked decrease in α-diversity indices (Shannon and Simpson), suggesting a loss of microbial diversity. After surgery, Bacteroidetes remained the dominant phyla, with a partial recovery of α-diversity indices, although remaining lower than at the initial stage. The β-diversity analysis, accessed by Bray-Curtis distance, corroborates these findings, with the most significant changes observed between the initial and intermediate timepoints, and a partial shift back in the final timepoint (data not shown). At the species level, there is a clear dominance of *Prevotella copri* in the intermediate and final timepoints (**Figure 4C**).

**Figure 5:**
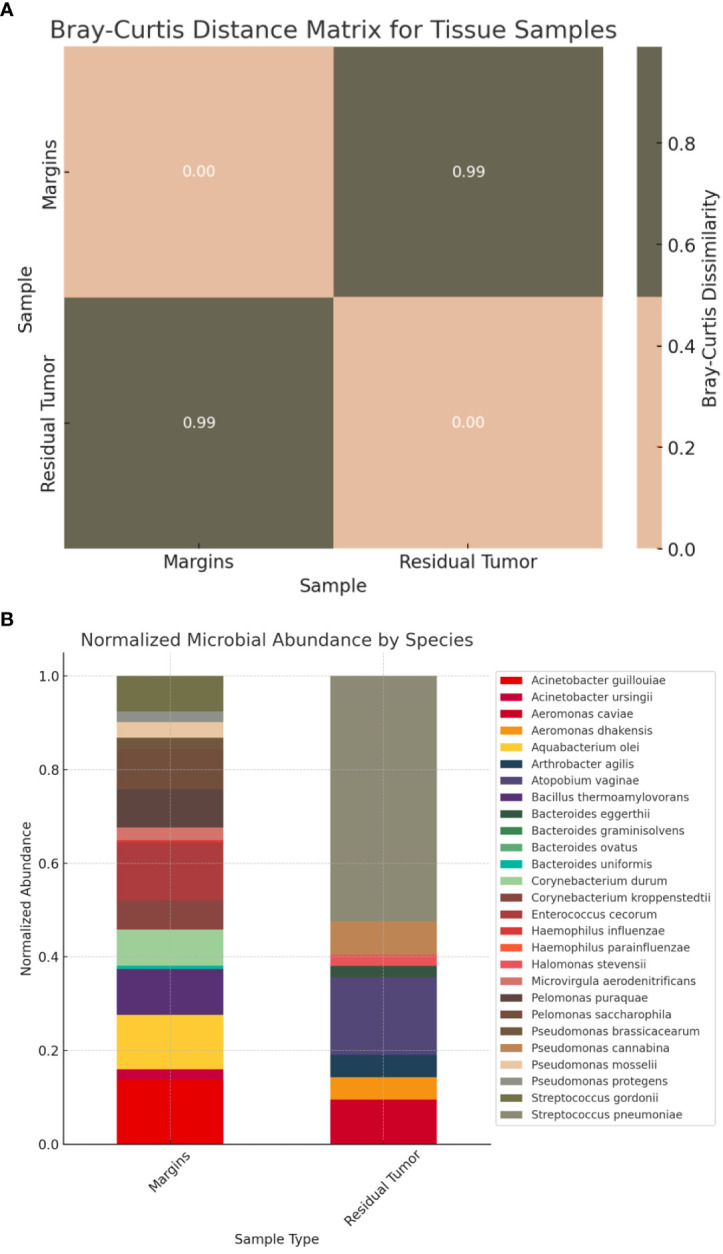
**(A)** Bray-Curtis distance matrix for tissue samples. The Bray-Curtis distance matrix shows a very high dissimilarity (approximately 0.995) between the two samples, suggesting that the microbial compositions of the residual tumor and respective margins are markedly different. **(B)** Relative abundance of microbial species in tissue samples (residual tumor and respective margins).

The analysis from the microbiota samples of the surgical specimen revealed an interestingly high dissimilarity between the residual tumor and respective margins, with statistical significance (*p*<0.001), suggesting markedly different microbial compositions (**Figure 5A**). While the margins revealed a more diverse distribution of microbial species, the tumor’s microbial composition was dominated by fewer species, particularly *Streptococcus pneumoniae* and *Atopobium vaginae* (**Figure 5B**).

## Discussion

This case allows for several interesting discussion points, namely the primary systemic therapy backbone selection, the available evidence supporting adjuvant therapy following a neoadjuvant approach with ET + CDK4/6i, and the microbiota analysis and its potential clinical implications.

### Endocrine therapy-based vs chemotherapy-based primary systemic therapy selection

In patients whose assessment by the multidisciplinary team is an inoperable breast primary tumor, it is essential to consider primary systemic treatment. The success of this treatment can determine the possibility of surgery with curative intent later in time. Chemotherapy (ChT) has been the mainstay of neoadjuvant treatment in HR+/HER2- breast cancer due to its higher pathological complete response (pCR) rates. However, pCR rates achieved in luminal-like tumors are still much lower than those observed in triple-negative or HER2+ breast cancer (for example, 14.9% vs. 41.9% vs. 55.1%) (21). At the same time, ChT is associated with an unfavorable toxicity profile that includes myelotoxicity, gastrointestinal toxicity, and skin disorders, among others, that can have serious short- or long-term impact. ET is a valid neoadjuvant option, although less considered than ChT, being usually reserved for patients who refuse or have contraindications to cytotoxic therapy and HR+ tumors (22).

The available evidence considering ET with or without CDK4/6i versus ChT in the neoadjuvant setting has been encouraging. Still, few trials and only phase II support this strategy for a limited set of patients (23).

The CTNeoBC pooled analysis showed that pCR was associated with better Event-free survival (EFS) in high-risk (G3) HR+/HER2- breast cancer (24), highlighting the importance of developing strategies to achieve higher pCR in this patient population. Two recently presented trials, KEYNOTE-756 (NCT03725059) and CheckMate 7FL (NCT04109066), used immunotherapy in an attempt to improve pCR rates and showed significantly improved pCR rates in the experimental arm of 24.3% and 24.5% vs. 15.6% and 13.8%, respectively (25, 26). EFS data were immature for both trials. Meanwhile, ET monotherapy has been reported to achieve pCR in 0–17.5% of cases (22). Of note, no adjuvant strategy is specifically dependent upon pCR in HR+/HER2- breast cancer, in contrast with triple-negative or HER2+ tumors.

Although obtained in a pre-and perimenopausal population, the phase II RIGHT Choice trial (NCT03839823) compared ribociclib + ET versus physician’s choice combination ChT in patients with aggressive HR+/HER2− advanced breast cancer, including inoperable locally advanced tumors like the case described (27). Patients treated with ribociclib + ET had a statistically significant and clinically meaningful PFS benefit of approximately 1 year, with similar overall response rates for both strategies. Furthermore, lower rates of treatment-related serious adverse events and discontinuation were seen in the ribociclib + ET group.

This patient’s tumor was considered primarily inoperable, and she was, therefore, proposed for primary systemic treatment. Following 6 months of ET + CDK4/6i, there was an almost 19% reduction in tumor size from 90 mm at baseline to 73 mm (using RECIST per MRIs measurements), a complete disappearance of the vegetation in the intermammary cleft, and a marked decrease in the metabolic profile on PET-FDG, which motivated a R0-intended surgery. However, the surgery was R1 due to the tumor’s posterior margin contact with the sternal periosteum, which precluded further margin extension. In fact, when analyzing the clinical-to-pathological downstaging of the tumor (cT4cN3M0 to ypT3ypN2aM0), it seems that the response achieved was far more modest than clinically assumed. Furthermore, the tumor’s Ki67 index remained untouched at 30% from the diagnostic biopsy to the surgical specimen. A high Ki67 index after neoadjuvant endocrine therapy is known to be a strong prognostic biomarker, being associated with worse recurrence-free survival and overall survival (28). Another interesting aspect in this case is the progesterone receptor downregulation following ET exposure, suggesting an *in vivo* selection of potentially ET-resistant clones.

### Adjuvant individualized decisions

After surgery, the patient’s case was rediscussed to define the adjuvant therapeutic plan. Adjuvant ET is an essential component of the treatment of HR+ breast cancer. AIs are the preferred adjuvant treatment for postmenopausal women, when compared to tamoxifen, with a favorable impact on recurrence and survival and a generally acceptable toxicity profile.

In the PENELOPE-B trial (NCT01864746), adjuvant palbociclib for 1 year in addition to ET did not improve invasive disease-free survival (iDFS) in women with residual invasive disease and at a high risk of relapse after taxane‐containing neoadjuvant ChT (29). This strategy was not applicable in this patient’s particular case since no ChT was used pre-surgically. The only CDK4/6i currently approved in adjuvancy is abemaciclib (150 mg orally twice a day for 2 years, in addition to ET) according to the monarchE trial that showed a sustained recurrence risk reduction (about 32%) in high-risk patients (7, 30). However, the monarchE trial excluded patients who had previously received treatment with CDK4/6i; therefore, no recommendation could be made for this patient based on this trial. Recently, the first data regarding the NATALEE trial (NCT03701334) were made available, supporting ribociclib plus ET in patients with stage II or III breast cancer (8). Prior (neo)adjuvant ET was allowed if initiated ≤ 12 months before randomization, but previous CDK4/6i use was an exclusion criterion, so, again, no recommendation can be made based on this trial. Therefore, despite this patient’s proposed adjuvant therapeutic plan, there is no current evidence supporting the use of adjuvant CDK4/6i in patients previously treated with CDK4/6i. Another important consideration is that, in this case, no surgical complications were potentially attributable to the previous use of ET plus CDK4/6i.

Olaparib, a poly (ADP-ribose) polymerase inhibitor (PARPi), has been approved in the adjuvant setting in high-risk BRCA1/2 mutated patients following the results of the OlympiA trial (NCT02032823) (31). Although the patient has not reported a family history consistent with breast-ovarian cancer syndrome and appears to have no personal criteria for genetic counseling, a BRCA1/2 germline mutation test could be useful to determine the benefit of adjuvant PARPi treatment. In this particular case, since the OlympiA trial only included patients treated with neo/adjuvant ChT, this evaluation was not considered useful at this stage, and with a low probability of mutation since there was no family history. Nevertheless, genomic testing is advised considering the patient’s high risk of recurrence since PARPi may constitute a future therapeutic option, according to EMBRACA (NCT01945775) and OlympiAD (NCT02000622) trials.

Regarding radiotherapy, being a locally advanced surgically removed R1 breast cancer, evidence strongly supports adjuvant radiotherapy.

### Microbiota insights and correlation with potential outcomes

#### Gut microbiota

Firmicutes and Bacteroidetes are the dominant phyla inhabiting the gut, accounting for approximately 90% of the entire gut microbiota (32). The shift from a Firmicutes-dominant to a Bacteroidetes-dominant profile in this patient’s gut microbiota across the therapeutic approach may have multifaceted implications.

The F/B ratio is known to have an important effect on maintaining gut homeostasis and is imbalanced in various health conditions (33). As an example, high F/B ratios have been seen in obesity, a recognized risk factor for breast cancer, though this association is still controversial (33, 34). A study conducted on 95 breast cancer patients showed that the F/B ratio was three times lower in patients with breast cancer in comparison to healthy controls (35). Luminal subtypes had higher F/B ratios than HER2+ or triple-negative breast cancers, and the ratio tended to decrease as cancer stage increased. The same study defined an optimal cutoff value for F/B ratio at 3.37, meaning that there is a break of gut microbial symbiosis and an increase in breast cancer risk below this value. Our patient had an F/B ratio at diagnosis of 2.73, thereby suggesting dysbiosis and increased risk for breast cancer.

Cancer therapy can influence microbiota composition as described in the case. Letrozole, for instance, has been shown to cause a time-dependent shift in gut microbiota in a mouse model (36). In that study, letrozole-treated mice evidenced different relative abundance of specific bacterial OTUs, most of them Bacteroidetes and Firmicutes phyla members, accompanied by a substantial reduction in overall species and phylogenetic richness. However, this relationship between microbiota and cancer therapy is not unidirectional. Changes in microbiota composition can also influence drug metabolism, thereby impacting cancer treatments’ efficacy and toxicity, a field known as pharmacomicrobiomics (14, 37).

Despite existing evidence on gut microbiota’s predictive utility in other tumor types, including HER2+ breast tumors, evidence in HR+/HER2- breast cancer is still scarce and preliminary (38, 39). A study conducted on 14 HR+/HER2- metastatic breast cancer patients recently addressed the potential relationship between gut microbiota and response to CDK4/6i (40). Although no significant differences were observed between responders and non-responders in terms of α-/β-diversity at the phylum or species level, four bacterial species were collectively able to predict response to CDK4/6i. The phyla analysis from that study shows a dominance of Firmicutes in both responder and non-responder cohorts, with F/B ratios of 2.7 and 2.1, respectively.

The Firmicutes-to-Bacteroidetes switch observed in our patient was mostly due to a noteworthy increase in the relative abundance of *Prevotella copri* following 6 months of letrozole and ribociclib. *Prevotella copri* is an abundant member of the human gut microbiota, whose relative abundance has curiously been associated with positive and negative impacts on several diseases, alongside some pharmacomicrobiomic implications (41). The link between *Prevotella copri* and different types of cancer remains inexplicit, although some hypothesize that Prevotella genera may be involved in breast disease due to its estrogen-deconjugating enzymatic activity (41, 42). The role of *Prevotella copri* and other bacterial species capable of metabolizing estrogens in breast cancer is a field of particular interest for future research.

Finally, a third dimension of the interaction between gut microbiota and cancer therapy comes from the observation that gut microbial shifts can influence gut health, which may greatly impact the patient’s quality of life through the gastrointestinal side effects often associated with cancer treatments (43).

This case report is unique because of the longitudinal analysis of the patient’s gut microbiota throughout the therapeutic approach with ET + CDK4/6i. Additional analyses would be of value in order to confirm the persistence of this microbial shift in the long term, namely after completing therapy with adjuvant letrozole or in case of recurrence. Unfortunately, such analyses are not possible due to BioBreast’s study protocol, thereby constituting a limitation to this case report.

#### Intratumoral microbiota

The analysis of the microbial composition of the residual tumor and respective margins revealed a high dissimilarity and differing dominant species between both samples, suggesting that they may come from different tissue conditions. These findings are aligned with a previous study that reported different microbial distributions between breast tumors and tumor-adjacent normal breast tissues (17). However, in contrast with that study, our analysis revealed a less diverse microbial population in the residual tumor in comparison with the respective margins, a finding that may be potentially related to the systemic therapy. The dominance of certain species in the residual tumor, like *Streptococcus pneumoniae* and *Atopobium vaginae*, might be of particular interest for further investigation in this field. A preclinical study showed that Streptococcus in breast cancer cells can inhibit the RhoA-ROCK signaling pathway to reshape the cytoskeleton and help tumor cells resist mechanical stress in blood vessels, thus promoting hematogenous metastasis (20). Although both samples were collected and conserved in similar conditions, the absence of direct controls may constitute a limitation to this preliminary analysis.

## Conclusions

This case provides an example of primary CDK4/6i + ET in locally advanced breast cancer considered primarily unresectable. This strategy allowed us to consider and perform a curative-intended surgery later in time. High-level evidence on the use of neoadjuvant CDK4/6i is highly awaited, but the increasing use of this approach will also raise more questions, namely on the potential implications on adjuvancy. Most adjuvant options currently available in HR+/HER2- breast cancer were approved based on trials that excluded patients previously treated with CDK4/6i, making decisions on adjuvancy potentially less evidence-based.

This case is also unique in that, as part of an investigational study, it reports an analysis of the patient’s gut microbiota throughout the disease course, something not currently performed in clinical practice. This analysis revealed a modulatory effect at this level following 6 months of ET + CDK4/6i. Future research might delve into how specific microbial alterations correlate with clinical outcomes and whether targeted microbiota modulation, such as probiotics or dietary interventions, might be employed as an adjuvant strategy in cancer management.

## Patient perspective

After multiple biopsies and exams, I was diagnosed with breast cancer in a slightly advanced stage. This diagnosis was the beginning of a 14-month journey. My first battle was taking ribociclib for 6 months. After that, I underwent surgery. I vividly remember waking up after the procedure with someone whispering in my ear that I had kept both breasts. I cannot express the immense happiness I felt upon hearing those words. Everything went down perfectly, and I am deeply grateful to my exceptional medical team for this success. Lastly, I had to endure more than 30 painful radiotherapy sessions, along with some physical therapy.

Despite the difficulties, everything ultimately turned out well. After these 14 months, I must extend my heartfelt thanks to my oncology team, who went above and beyond to ensure a positive outcome. I fought for my will to live and held onto my faith that everything would turn out well.

## Data availability statement

All relevant data is contained within the article: The original contributions presented in the study are included in the article/**Supplementary Material**, further inquiries can be directed to the corresponding authors.

## Ethics statement

The studies involving humans were approved by The ethics committee of the CUF Descobertas Hospital. The studies were conducted in accordance with the local legislation and institutional requirements. The participants provided their written informed consent to participate in this study. Written informed consent was obtained from the individual(s) for the publication of any potentially identifiable images or data included in this article.

## Author contributions

GV: Writing – original draft, Writing – review & editing. DC: Conceptualization, Formal analysis, Funding acquisition, Investigation, Methodology, Project administration, Resources, Supervision, Validation, Visualization, Writing – review & editing. MF-S: Supervision, Writing – review & editing. PCR: Data curation, Formal analysis, Investigation, Methodology, Resources, Software, Writing – review & editing, Validation, Visualization. FM: Resources, Supervision, Writing – review & editing. CBS: Supervision, Writing – review & editing. CRS: Supervision, Writing – review & editing. IN: Writing – review & editing, Supervision. AC: Methodology, Validation, Writing – review & editing. PB: Methodology, Supervision, Validation, Writing – review & editing. AGP: Methodology, Supervision, Validation, Writing – review & editing. CM: Supervision, Validation, Writing – review & editing, Methodology. JGS: Supervision, Writing – review & editing, Resources. RC: Investigation, Supervision, Writing – review & editing. JCR: Investigation, Supervision, Validation, Writing – review & editing. CC: Investigation, Project administration, Supervision, Validation, Visualization, Writing – review & editing. AF: Formal analysis, Investigation, Methodology, Project administration, Supervision, Validation, Visualization, Writing – review & editing.

## Supplementary material

Graphic 1
**(A)** Firmicutes/Bacteroidetes (F/B) ratio in gut microbiota across timepoints. The F/B ratio decreases from 2.73 at the initial timepoint (diagnosis) to 0.13 at the intermediate timepoint (following 6 months of systemic therapy) and 0.15 at the final timepoint (following surgery), reflecting a major shift from a Firmicutes-dominant profile at the initial timepoint to a Bacteroidetes*-*dominant profile in the subsequent timepoints. **(B)** α-diversity indices in gut microbiota across timepoints. At diagnosis, Shannon’s and Simpson’s diversity indices were 2.58 and 0.89, respectively, indicating a high diversity at this stage. There is a significant decrease in both indices at the intermediate timepoint (0.58 and 0.20), followed by a partial recovery by the final timepoint (1.17 and 0.53). **(C)** Relative abundance of microbial species in gut microbiota across timepoints.

Graphic 2
**(A)** Bray-Curtis distance matrix for tissue samples. The Bray-Curtis distance matrix shows a very high dissimilarity (approximately 0.995) between the two samples, suggesting that the microbial compositions of the residual tumor and respective margins are markedly different. **(B)** Relative abundance of microbial species in tissue samples (residual tumor and respective margins).
