# Supplementary material for: Case report: Primary CDK4/6 inhibitor and endocrine therapy in locally advanced breast cancer and its effect on gut and intratumoral microbiota
Source: Front Oncol. 2024 Mar 27;14:1360737. doi: 10.3389/fonc.2024.1360737 (PMC11004348; doi:10.3389/fonc.2024.1360737)
Supplement: Graphic 1 — (A) Firmicutes/Bacteroidetes (F/B) ratio in gut microbiota across timepoints. The F/B ratio decreases from 2.73 at the initial timepoint (diagnosis) to 0.13 at the intermediate timepoint (following 6 months of systemic therapy) and 0.15 at the final timepoint (following surgery), reflecting a major shift from a Firmicutes-dominant profile at the initial timepoint to a Bacteroidetes-dominant profile in the subsequent timepoints. (B) α-diversity indices in gut microbiota across timepoints. At diagnosis, Shannon’s and Simpson’s diversity indices were 2.58 and 0.89, respectively, indicating a high diversity at this stage. There is a significant decrease in both indices at the intermediate timepoint (0.58 and 0.20), followed by a partial recovery by the final timepoint (1.17 and 0.53). (C) Relative abundance of microbial species in gut microbiota across timepoints. [file DataSheet_1.docx]

**Supplementary material**

**Fecal sample collection and management**

Fecal samples were collected using an EasySampler^®^ Stool Collection Kit (ALPCO) provided by the research team and the patient had to deliver the collection kit within 48 hours after harvesting. Stool samples were then maintained in RNAlater (Sigma) and stored at -80^o^C until analysis.

**Intratumoral microbiota collection and management**

For intratumoral microbiota analysis, frozen breast tissue sections (15–20 at 4 μm) were cut from each block and stored at -80°C prior to DNA extraction. DNA extraction was done using a DNA Isolation Kit. DNA concentrations were measured and samples with sufficient DNA were enriched for microbial DNA using a Microbiome DNA Enrichment Kit.

**Microbiota analysis**

Genomic DNA was extracted from the samples using a reliable extraction method with focus on the V3 and V4 regions of the 16S rRNA gene, which were amplified using the "Ion 16S™ Metagenomics Kit". The amplified sequences were then subjected to high-throughput sequencing on an advanced platform, providing detailed insights into the microbial composition. Initial sequencing data processing involved quality checks and filtering to remove low-quality sequences, ensuring the integrity of the subsequent analysis. The DADA2 pipeline was employed for error correction and the generation of Amplicon Sequence Variants (ASVs), enabling high-resolution identification of microbial taxa. For taxonomic classification, the ASVs were mapped against the SILVA database (version 138), a comprehensive reference for microbial identification. Data organization and integration were facilitated by Phyloseq.

Key analyses included calculating each sample’s Firmicutes/Bacteroidetes ratio, given its significance in evaluating microbial balance and potential dysbiosis. The relative abundance of microbial species across samples was analyzed to understand the diversity and predominant taxa. To assess the variations in microbial communities between samples, Bray-Curtis dissimilarity analysis was conducted. To access statistically significant differences in the bacterial composition of gut and intratumoral microbiota, the Kruskal-Wallis H-test was employed for a statistical significance of *p*<0.05. These analyses were performed using a combination of Python and R programming languages, leveraging libraries such as Pandas for data manipulation, and Seaborn and Matplotlib for graphical visualizations.
